# Zfp322a Regulates Mouse ES Cell Pluripotency and Enhances Reprogramming Efficiency

**DOI:** 10.1371/journal.pgen.1004038

**Published:** 2014-02-13

**Authors:** Hui Ma, Hui Min Ng, Xiuwen Teh, Hu Li, Yun Hwa Lee, Yew Mei Chong, Yuin Han Loh, James J. Collins, Bo Feng, Henry Yang, Qiang Wu

**Affiliations:** 1 Department of Biochemistry, Yong Loo Lin School of Medicine, National University of Singapore, Singapore; 2 Wyss Institute for Biologically Inspired Engineering, Harvard University, Boston, Massachusetts, United States of America; 3 Howard Hughes Medical Institute, Boston, Massachusetts, United States of America; 4 Institute of Molecular and Cell Biology, Proteos, Singapore; 5 Department of Biological Sciences, National University of Singapore, Singapore; 6 Department of Biomedical Engineering and Center for BioDynamics, Boston University, Boston, Massachusetts, United States of America; 7 School of Biomedical Sciences, The Chinese University of Hong Kong, Shatin, New Territories, Hong Kong; 8 Cancer Science Institute of Singapore, Centre for Translational Medicine, Singapore; University of Illinois, United States of America

## Abstract

Embryonic stem (ES) cells derived from the inner cell mass (ICM) of blastocysts are characterised by their ability to self-renew and their potential to differentiate into many different cell types. Recent studies have shown that zinc finger proteins are crucial for maintaining pluripotent ES cells. Mouse zinc finger protein 322a (Zfp322a) is expressed in the ICM of early mouse embryos. However, little is known regarding the role of Zfp322a in the pluripotency maintenance of mouse ES cells. Here, we report that Zfp322a is required for mES cell identity since depletion of *Zfp322a* directs mES cells towards differentiation. Chromatin immunoprecipitation (ChIP) and dual-luciferase reporter assays revealed that Zfp322a binds to *Pou5f1* and *Nanog* promoters and regulates their transcription. These data along with the results obtained from our ChIP-seq experiment showed that Zfp322a is an essential component of mES cell transcription regulatory network. Targets which are directly regulated by Zfp322a were identified by correlating the gene expression profile of *Zfp322a* RNAi-treated mES cells with the ChIP-seq results. These experiments revealed that Zfp322a inhibits mES cell differentiation by suppressing MAPK pathway. Additionally, Zfp322a is found to be a novel reprogramming factor that can replace Sox2 in the classical Yamanaka's factors (OSKM). It can be even used in combination with Yamanaka's factors and that addition leads to a higher reprogramming efficiency and to acceleration of the onset of the reprogramming process. Together, our results demonstrate that Zfp322a is a novel essential component of the transcription factor network which maintains the identity of mouse ES cells.

## Author Summary

Embryonic stem (ES) cells are featured by their ability to self-renew and by their potential to differentiate into many different cell types. Recent studies have revealed that the unique properties of mouse ES cells are governed by a specific transcription regulatory network, including master regulators Oct4/Sox2/Nanog and other pluripotency factors. The importance of these factors was highlighted by the subsequent finding that combination of several transcription factors can reprogram differentiated fibroblasts back to pluripotent stem cells. Here, we report that Zfp322a is a novel factor which is required for mES cell identity. We revealed that Zfp322a can regulate the key pluripotency genes *Pou5f1* and *Nanog* and functions as a repressor of MAPK/ERK pathway in mES cells, therefore preventing mES cell differentiation. Furthermore, we discovered that Zfp332a can promote the generation of induced pluripotent stem cells (iPSCs) from mouse embryonic fibroblasts (MEFs). Our results reveal that Zfp322a is a novel essential transcription factor which not only regulates ES cell pluripotency but also enhances iPSC formation.

## Introduction

Embryonic stem (ES) cells, which are derived from the inner cell mass (ICM) of mammalian blastocysts, are characterised by their ability to self-renew and by their potential to differentiate into many different cell types [1], [2]. ES cells provide a prime platform for biomedical research since the investigation of factors and pathways that control pluripotency and differentiation provides us with valuable data that will aid in the advancement of regenerative [3]. The discovery that differentiated cells can be reprogrammed into induced pluripotent stem cells (iPSCs) gave great promises for the progress of regenerative medicine and gene therapy [4]–[7].

It has been discovered that transcription factors play crucial roles in controlling ES cell identity. Genome-wide analyses revealed that in mammalian ES cells, Oct4, Nanog and Sox2 form the core transcriptional circuitry that activate genes involved in self-renewal and pluripotency and repress genes that promote differentiation into different lineages [8], [9]. The importance of this transcription network was subsequently highlighted by the finding that the expression of just four transcription factors, Oct4, Sox2, c-Myc and Klf4 (OSKM) was sufficient to transform mouse embryonic fibroblasts (MEFs) back to pluripotent stem cells, and the expression of OCT4, SOX2, NANOG and LIN28 was sufficient for in human somatic cell reprogramming [6], [7]. Along with these core factors, there are many other transcription factors which closely interact with these factors (i.e. form the gene regulatory network). Hence it is important to unravel the functions of all components in order to fully understand how this regulatory network functions to regulate various target genes. Therefore, it is of great value to expand our knowledge of this transcription regulatory network.

Krüeppel associated box (KRAB) C2H2 zinc finger family can interact directly with specific cis-regulatory DNA elements to regulate genes' activities [10]. Several studies have revealed that proteins from this family, such as ZSCAN4, Zfp296, Zfp206, and Zfp42, are key components of the ES transcriptional network and are crucial for maintaining pluripotent ES cells [11]–[15]. Mouse zinc finger protein 322a (Zfp322a) is another evolutionarily conserved protein that belongs to this family [16]. We proposed that Zfp322a acts as a transcription factor in mouse ES cells for two main reasons: First, ChIP-seq data from a previous study suggested that pluripotency factors Oct4, Zfx, E2F1, Klf4 and Myc bind to the genomic region of *Zfp322a* [17]. Second, Zfp322a is expressed at a higher level in mES cells compared to trophoblast cells [18]. Additionally, single cell RNA-seq results have demonstrated *Zfp322a* expression in both mouse ICM and ES cells [19]. These results suggested a potential role for Zfp322a in controlling the identity of mES cells. However, there has been little information regarding the function of Zfp322a.

In this study, we demonstrated that Zfp322a is required for the maintenance of mouse ES cell identity. Depletion of *Zfp322a* impairs mES cell self-renewal and induces them to differentiate. We found that Zfp322a positively regulates *Pou5f1* and *Nanog* expression, and possibly represses MAPK/ERK pathway, thus preventing mES cells from differentiation. Further genome wide studies also identified the targets of Zfp322a which are involved in a variety of biological processes, including DNA transcription and translation, chromosome organization, development, DNA repair, cell cycle and apoptosis. Through iPSC formation assays we discovered that Zfp322a can be used as a novel reprogramming factor and the reprogramming efficiency was enhanced by the addition of Zfp322a to OKSM. Together, these results established Zfp322a as a novel pluripotency factor that can enhance reprogramming process.

## Results

### 1. Zfp322a is required for the maintenance of mES cell self-renewal and pluripotency

Chen *et al.* demonstrated that Oct4, Zfx, E2F1, Klf4 and Myc bound to an 800 bp-region in the third intron of *Zfp332a* [17] (Figure S1A). Our ChIP results confirmed the interaction between Oct4 and *Zfp322a* intron (Figure S1B). This suggested that *Zfp322a* is a direct target of regulation by these transcription factors. In previous studies Zfp322a was discovered to be expressed at a higher level in ICM compared to trophectoderm [18]. We further examined the expression of Zfp322a in mES cells by immunostaining assay (Figure S2A), using the antibody specific for Zfp322a protein (Figure S2B). We found that Zfp322a was mainly localized in the nucleus of mES cells, which indicates that as a zinc finger protein, Zfp322a may function as a transcription factor in mES cells.

**Figure 1:**
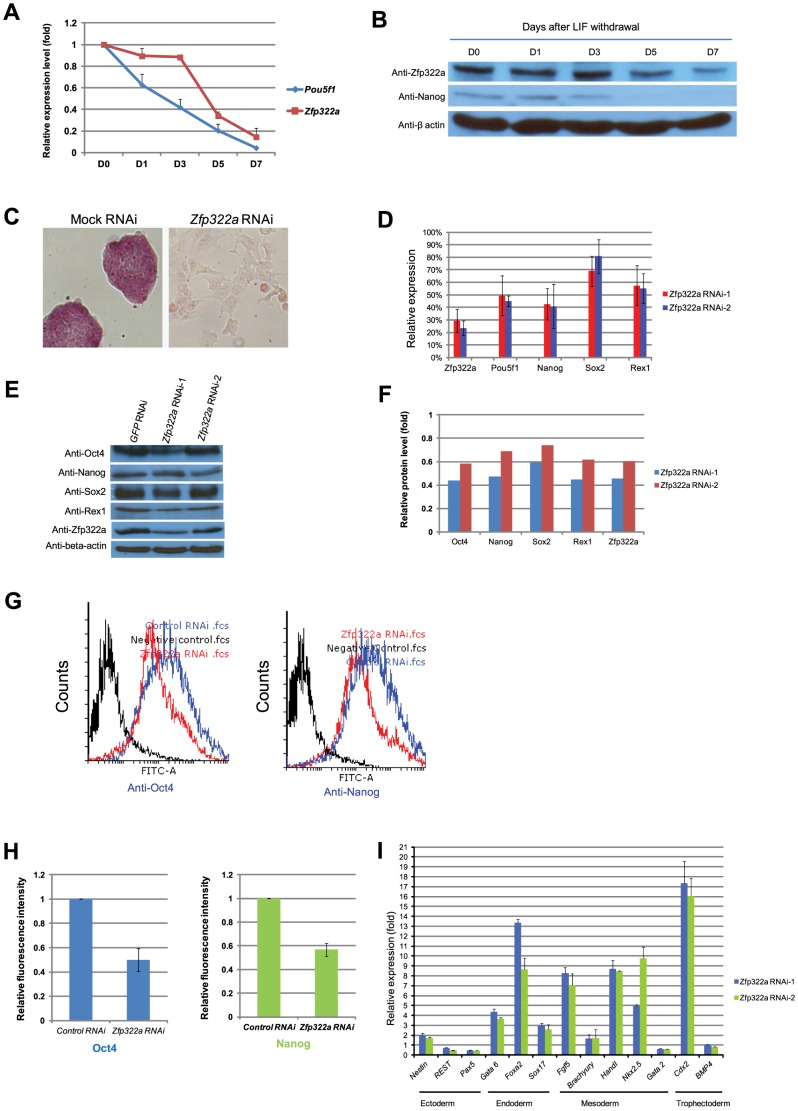
Zfp322a is required for ES pluripotency. (**A**) *Zfp322a* mRNA level was decreased in mES cells cultured in LIF withdrawal ESC medium. The level of the *Zfp322a* and *Pou5f1* mRNA were compared to control cells cultured in normal ESC medium and normalized against *β-actin*. (**B**) Zfp322A protein level showed a reduction as mES cells differentiated. β-actin served as loading control. (**C**) *Zfp322a* RNAi caused ES cell differentiation. AP staining was conducted on the fourth day of selection after the cells were transfected with *Zfp322a* shRNAs. *Zfp322a* RNAi cells displayed a lighter colour compared to the dark red colour of mock RNAi cells. (**D**) Pluripotency genes were down-regulated upon *Zfp322a* RNAi. ES cells transfected with empty pSUPER.puro vector were used as a control and gene expression levels were normalized against *β-actin*. (**E**) *Zfp322a* RNAi resulted in decreased Oct4, Nanog, Sox2 and Rex1 protein. β-actin served as loading control. (**F**) Quantification of the protein level changes. Protein levels of *Zfp322a* knocked-down cells were normalized against β-actin and compared to control RNAi cells using software ImageJ. (**G**) Representative flow cytometry results showed that fluorescence intensities of Oct4 and Nanog were repressed in *Zfp322a* depleted cells as compared to control cells. (**H**) *Zfp322a* depletion led to reductions of Oct4 and Nanog expression at cellular levels. The mean of fluorescence intensity was calculated using software flow software 2.5.0. Relative fluorescent intensities of *Zfp322a* RNAi cells were normalized against control knocked-down cells. Standard deviations were derived from three independent experiments. (**I**) *Zfp322a* RNAi caused up-regulation of lineage specific markers for endoderm, mesoderm and trophectoderm. Specific primers were used to check the respective gene expression levels by real-time PCR.

To determine the change in the expression of Zfp322a upon mES cell differentiation, mouse ES cells were induced to differentiate by culturing in LIF (leukemia inhibitory factor) withdrawal medium. *Zfp322a* mRNA level was reduced during the differentiation process, dropping to 20% at 7 days after LIF removal (Figure 1A). Similarly, Zfp322a protein also decreased upon mES cell differentiation (Figure 1B). The expression of Zfp322a in undifferentiated mES cells and its repression upon ES cell differentiation further suggested a possible involvement of Zfp322a in pluripotency maintenance in mouse ES cells.

In order to investigate the role of Zfp322a in mouse ES cell pluripotency, we examined the effect of *Zfp322a* depletion in mES cells by RNAi. E14 mES cells were transfected with two independent *Zfp322a* shRNAs targeting different regions of *Zfp322a* gene. Both shRNAs were effective in depleting the level of *Zfp322a* mRNA to 30% of the control (Figure 1D). Upon knock-down of *Zfp322a*, mES cells lost their characteristics, including the round colony-like morphology and alkaline phosphatise (AP) activity. Instead, RNAi-treated cells exhibited flattened, differentiated cell morphology (Figure 1C). These results indicated that *Zfp322a* depletion caused mES cell differentiation and impaired self-renewal of mES cells.

### 2. Depletion of Zfp322a activates developmental genes while repressing pluripotency related genes

We further examined the alteration in gene expression induced by *Zfp322a* depletion. The mRNA levels of pluripotency genes *Pou5f1*, *Nanog*, *Sox2* and *Zfp42* were significantly reduced in RNAi-treated E14 cells (Figure 1D). Consistently, protein levels of these pluripotency factors were also reduced upon *Zfp322a* depletion (Figure 1E, 1F). In addition, immunofluorescence (IF) of Oct4 and Nanog were performed to examine their expression at cellular level. The fluorescence intensities of *Zfp322a* knocked-down cells and control cells were measured with flow cytometry. Upon *Zfp322a* RNAi, there was a significant reduction of the fluorescence intensities observed both in anti-Oct4 antibody and anti-Nanog antibody stained cells (Figure 1G). Further we examined the population mean value of fluorescence intensities. We found that *Zfp322a* depletion supressed Oct4 and Nanog IF mean intensities by 50%, 40% respectively as compared to control (Figure 1H). Similar results were obtained in another mES cell line HM1 (Figure S5A). Since Oct4 and Nanog are essential for maintenance of pluripotency, these results are consistent with the observation that *Zfp322a* RNAi induces differentiation of mES cells. The differentiation of mES cells was further supported by the dramatic increase in various lineage markers after *Zfp322a* depletion (Figure 1I). Upon knock-down of *Zfp322a* in mES cells, we observed an up-regulation of endodermal markers: *Gata6* (4 fold), *Foxa2* (8 fold) and *Sox17* (3 fold), which indicated that Zfp322a could maintain mES cell pluripotency by repressing endodermal specification. Trophectoderm maker *Cdx2* displayed a 16 fold increase while mesoderm markers, *Fgf5*, *Hand1* and *Nkx2.5* increased by 7, 8 and 10 fold respectively (Figure 1I). Thus consistent with the AP staining results, this suggested that Zfp322a is required to suppress lineage specific gene expressions to maintain mES cells in their undifferentiated state.

**Figure 2:**
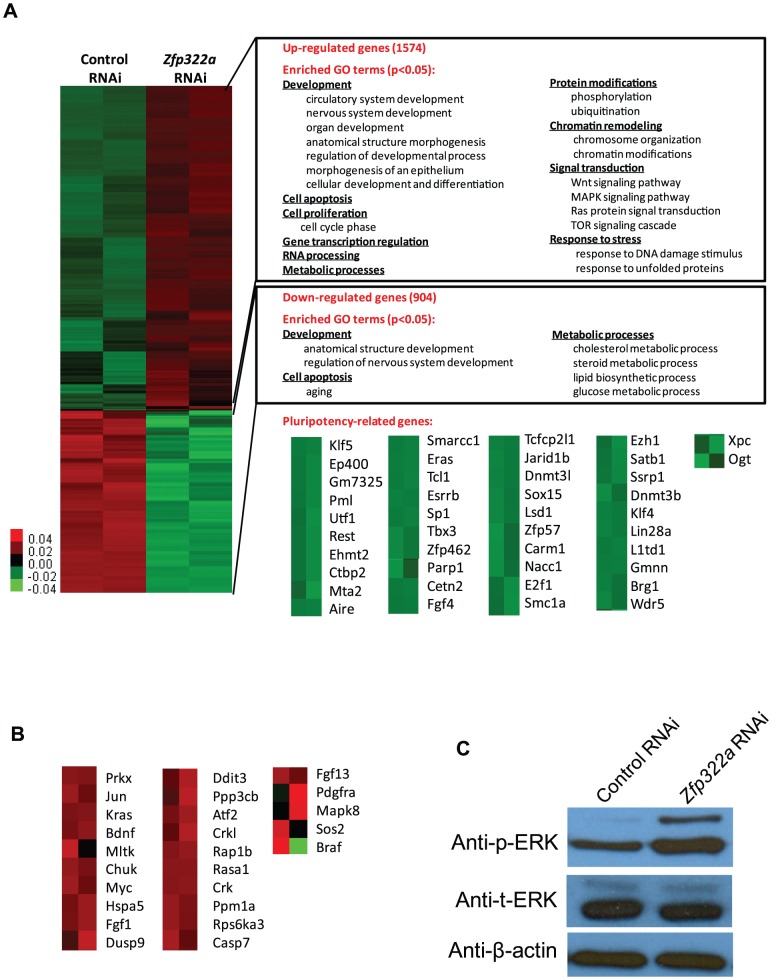
Changes of global gene expression upon *Zfp322a* knock-down in ES cells. (**A**) Microarray heat map generated from relative gene expression levels. Zfp322a was knocked down in E14 cells and the cells were selected for 96 hours before whole genome cDNA microarray hybridization was performed. Duplicates were performed to ensure reproducibility of results. Relative highly expressed genes were shown in red and low expressed genes in green. 1574 genes showed an increased expression level of more than 1.5 fold after *Zfp322a* RNAi. Gene onthology analysis was performed relating to “biological process”. The enriched terms were classified into several function groups and listed in the figure. Many terms related to developmental processes were enriched. 904 genes expression level showed more than 1.5 fold reduction. Examples of down-regulated pluripotency-related genes upon *Zfp322a* knock-down in ES cells. Genes were selected according to their known functions in pluripotency or ES cells. Each selected gene was taken as individual tiles from the thumbnail-dendogram duplicates. (**B**) List of up-regulated MAPK pathway related genes upon *Zfp322a* RNAi. Genes were selected as they fell into the cluster “MAPK signaling pathway” according to gene ontology analysis for enriched KEGG pathways. Each selected gene was taken as individual tiles from the thumbnail-dendogram duplicates. (**C**) Phosphorylated ERK (p-ERK) level was elevated in *Zfp322a* depleted cells as compared to control cells, while the total ERK (t-ERK) level was not affected. β-actin served as a control for normalization.

To further understand how *Zfp322a* depletion led to mES cell differentiation, we used gene expression microarrays to investigate the global gene expression profile changes induced by *Zfp322a* depletion (Figure 2A). qPCR experiments were performed to validate the results of the microarray analysis (Figure S3). As an internal control, we examined the level of *Zfp322a* by the microarray, and consistent with qPCR results (Figure 1D), we found an approximately 3-fold reduction in the mRNA level of *Zfp322a*. Upon *Zfp322a* depletion, 1574 genes were up-regulated (increased by >1.5 fold) and 904 genes were down-regulated (reduced by >1.5 fold) (Figure 2A). Importantly, the microarray data analysis revealed that many known pluripotency genes were down-regulated (Figure 2A). This indicated that Zfp322a is a high-level regulator in the mES cell gene regulatory network, which does not only regulate a subset of genes required for pluripotency, but is an essential component of the core network required for the maintenance of mES cell identity.

To determine whether Zfp322a regulated specific types of gene, we conducted gene ontology (GO) analysis of up-regulated and down-regulated genes. The enriched terms were summarized in Figures 2A (Full list of the enriched terms can be found in Table S1.). For the up-regulated genes, many terms related to development were enriched. This is consistent with the role of Zfp322a as a repressor of differentiation. Furthermore, *Zfp322a* depletion activated cell apoptosis related genes, also explained the increased apoptosis in *Zfp322a* RNAi-treated cells. Interestingly, many terms were related to chromosome remodelling, suggesting that Zfp322a may contribute to maintenance of the unique mES chromatin structure. Notably, several signaling pathways implicated in pluripotency such as MAPK pathway, Wnt signaling pathway, Ras signal cascade were also affected after Zfp322a depletion (Table S1, S2, Figure 2A, 2B). Importantly, *Zfp322a* depletion caused an up-regulation of phosphorylated ERK (p-ERK) level while the total levels of ERK (t-ERK) expression was not affected (Figure 2C). This substantiated that Zfp322a indeed is implicated in the repression of MAPK/ERK cascade without changing ERK expression in mES cells (Figure 2C).

It has been shown that inhibition of MAPK/ERK pathway is important for mES ground state pluripotency [20], [21]. Activation of Ras-MAPK pathway promotes trophectoderm induction and is specifically suppressed by Oct4 in undifferentiated mES cells [22]. ERK pathway has been found to promote the onset of mES cell differentiation and it is involved in multiple developmental processes [23]. Therefore, Zfp322a may maintain mES cell in undifferentiated state via repression of the MAPK/ERK cascade.

To further investigate the role of Zfp322a in MAPK pathway, we next examined whether inhibition of the MAPK pathway could rescue the effects caused by *Zfp322a* depletion. *Zfp322a* depleted mES cells were subjected to 50 nM, 250 nM and 1 uM of ERK inhibitors (PD0325901, Sigma). We found that the addition of ERK inhibitors could not rescue the down-regulated *Pou5f1*, *Sox2* and *Zfp42* upon *Zfp322a* depletion, and the ERK inhibitor treated cells exhibited differentiated morphology same as DMSO treated control cells (Figure S3B). However, with the addition of ERK inhibitors, levels of *Nanog, Sox2 and Rex1* were higher than that of *Zfp322a* depleted cells without ERK inhibitor and the endoderm and ectoderm lineage markers were lower (Figure S3B, S3C). This is consistent with previous findings in which the blocking of ERK pathway induces elevated expression of Nanog in ES cells [24], [25]. However, ERK inhibitor did not rescue stem cell differentiation caused by *Zfp322a* knocked-down cells (Figure S3B, S3C). Interestingly, addition of ERK inhibitors seemed to facilitate *Zfp322a* knocked-down cells to differentiate into mesoderm lineage, other than endoderm or ectoderm lineages cells (Figure S3B, S3C). This is in consistent with previous finding that *Erk2*-null mES cells tend to differentiate to mesoderm lineage more efficiently than wild type mES cells [25].

Taken together, it appears that *Zfp322a* depletion leads to activation of MAPK/ERK pathway, which could drive mES cells towards differentiation. However, the inhibition of MAPK/ERK pathway could not rescue the differentiation phenotype caused by *Zfp322a* loss.

### 3. Transcriptional regulation of *Pou5f1* and *Nanog* by Zfp322a

**Figure 3:**
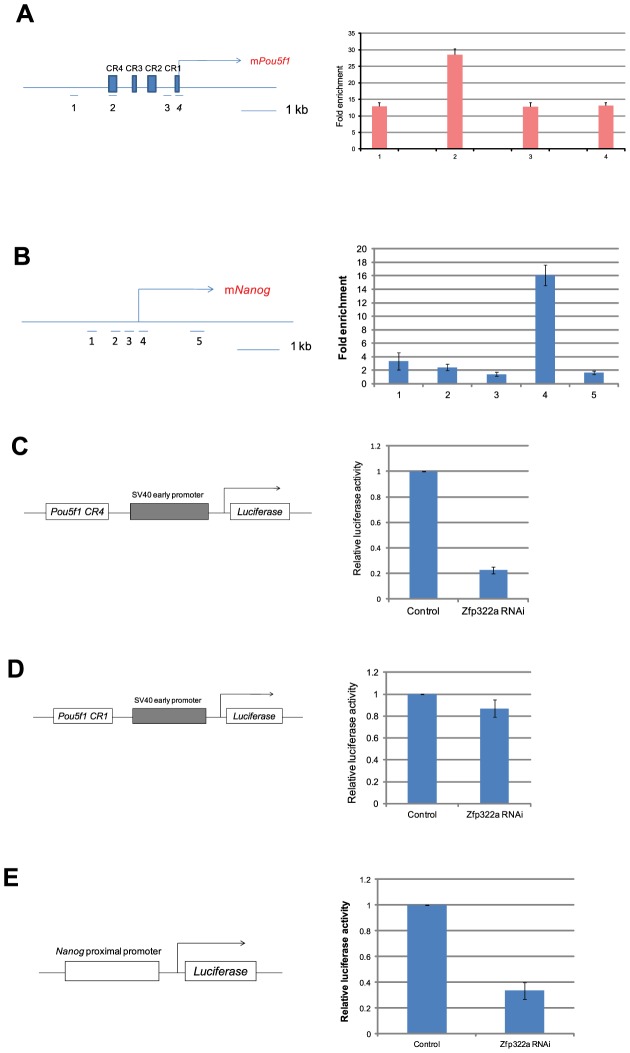
Zfp322a positively regulates *Oct4* and *Nanog* transcription. (**A**) Zfp322a binds to *Oct4* distal enhancer regions. Zfp322a ChIP DNA was analyzed by real-time PCR. Locations of primers used in qPCR were mapped to the *Pou5f1* genomic region. (**B**) Zfp322a binds to *Nanog* proximal promoter, with a highest enrichment fold at TSS starting site. Locations of primers were pictured on mouse *Nanog* genomic region. Relative luciferase activities were down-regulated upon *Zfp322a* RNAi using *Pou5f1* CR4-pSV40-*Luc* construct (**C**) and p*Nanog* PP-*Luc* construct (**E**), but not *Pou5f1* CR1-pSV40-*Luc* construct (**D**). Schematic structures of the constructs were presented. Empty pSUPER.puro vector were transfected in ES cells as a control RNAi. Renilla luciferase vector were transfected simultaneously and relative luciferase activities were normalized against Renilla luciferase activity.

Oct4 and Nanog are master regulators of mES pluripotency [26], [27]. Many pluripotency factors were found to bind to promoters of *Pou5f1* and *Nanog* to regulate their transcriptions [17]. Since *Nanog* and *Pou5f1* were down-regulated upon *Zfp322a* depletion, we speculated that Zfp322a may bind to *Pou5f1* and *Nanog* promoters to regulate their transcription. To test whether Zfp322a binds to cis-regulatory elements of *Pou5f1* and *Nanog*, ChIP experiments were performed using an anti-Zfp322a antibody to pull-down wild type mES cell chromatin. Real-time PCR was used to determine whether Zfp322a preferentially bound to known enhancer elements upstream of *Pou5f1* and *Nanog* promoters. We found a clear peak in the *Pou5f1* distal enhancer, which is also known as CR4 (conserved region 4), showing a 28 fold enrichment (Figure 3A, S5B). CR4 is the main enhancer that drives *Pou5f1* expression in mouse ES cells and early embryos, and it is the site bound by many transcription factors, including Nanog, Sox2 and Oct4 itself [8], [28], [29]. Similarly, Zfp322a was also shown to bind to the *Nanog* proximal promoter. Strong enrichment for amplicon 4 was found in the multiple transcription factor binding locus of *Nanog* promoter (MTL) (Figure 3B, S5C) [13]. These results showed that Zfp322a could directly bind to *Pou5f1* distal enhancer and *Nanog* proximal promoter in mES cells and may cooperate with other transcription factors in the regulation of *Pou5f1* and *Nanog* transcription.

To determine whether Zfp322a regulates the transcription of *Pou5f1* and *Nanog*, dual-luciferase assays were performed using two constructs *Pou5f1* CR4-pSV40-*Luc* and p*Nanog* pp-*Luc*. Interestingly, upon knock-down of *Zfp322a*, the luciferase activities were strikingly reduced to 20% and 30% respectively in constructs carrying the CR4 or the *Nanog* proximal promoter (Figure 3C, 3E, S5D). To determine whether this reduction was led by *Zfp322a* loss directly, *Pou5f1* CR1 was chosen as negative control. ChIP experiments showed a relatively lower enrichment fold at this region as compared to CR4. As expected, in the experiment with *Pou5f1* CR1-pSV40-*Luc* construct, the luciferase activity was only reduced by 15% upon *Zfp322a* depletion, much less than 80% reduction observed in the CR4 experiment (Figure 3D). These strongly suggested that Zfp322a directly regulated *Pou5f1* and *Nanog* through binding to these cis-regulatory elements. Interestingly, it was also observed that compared to single knock-down of *Pou5f1*, double knock-down of *Pou5f1* and *Zfp322a* further suppressed enhancer activities, (Figure S4A, S4B). Given that Oct4 also binds to CR4 and *Nanog* MTL to regulate the transcriptions [8], we hypothesized that Zfp322a may cooperate with Oct4 to regulate gene transcriptions.

### 4. Genome-wide mapping of Zfp322a binding sites in mES cells

To gain more insights into the downstream pathways through which Zfp322a functions, we identified genome-wide binding sites of Zfp322a in mouse ES cells. Following chromatin immunoprecipitation using anti-Zfp322a antibody to enrich the DNA fragments bound by Zfp322a, we used high-throughput sequencing (ChIP-seq) to analyze the ChIP-enriched DNA. Genomic regions defined by multiple overlapping DNA fragments derived from the ChIP enrichments were considered as putative binding sites. To confirm the validity of these putative binding sites, genomic loci with peaks of various fold changes were arbitrarily selected and tested by qPCR. The final threshold value was determined based on enrichment of 2 fold in qPCR validation (Figure S6), which corresponded to 9-fold or higher enrichment in the ChIP-seq experiment. This gave a total of 4382 putative binding sites of Zfp322a that were associated with 4056 genes (Table S3).

**Figure 4:**
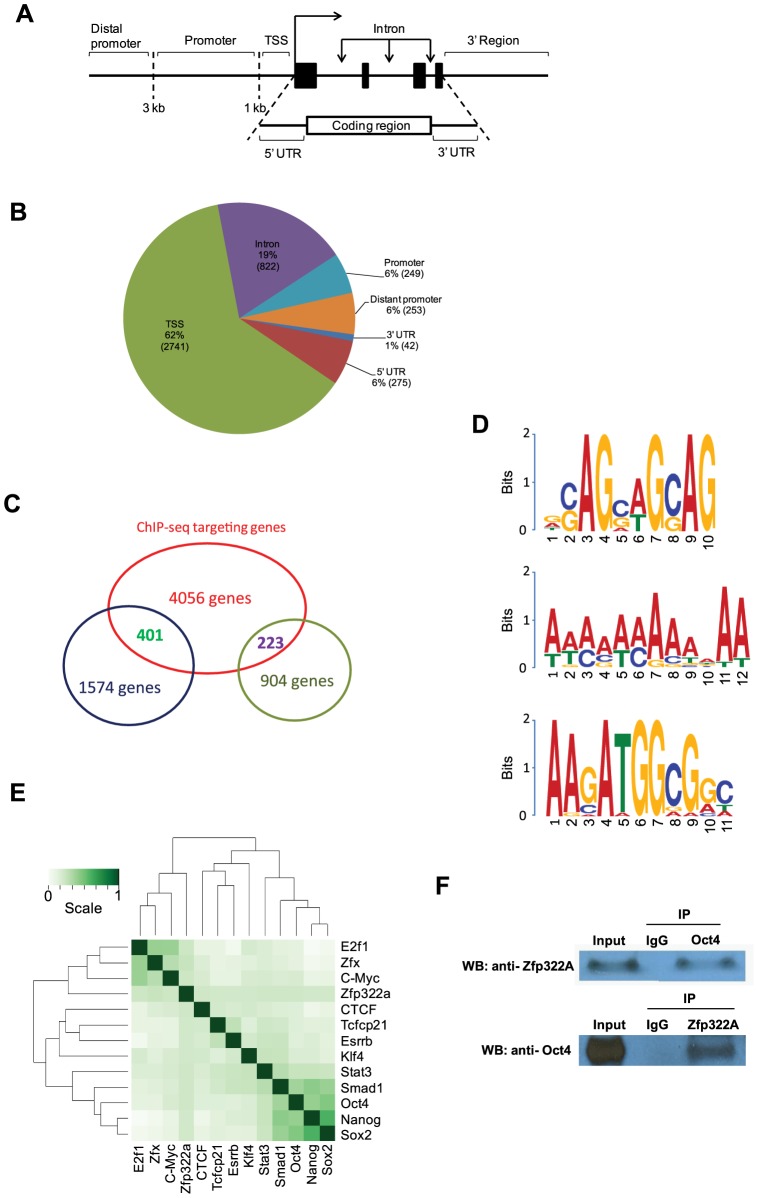
Genomic-wide analysis of Zfp322a binding sites. (**A**) Schematic definitions of locations of the putative Zfp322a binding sites relative to the nearest transcriptional unit. TSS referred to −1000 to +100 bp from 5′-end of annotated RNA. (**B**) Genomic distributions of Zfp322a binding loci. (**C**) Identification of genes that were predicted to be directly regulated by Zfp322a. The datasets from microarray analysis and ChIP-Seq targets were calculated for overlapping genes. The results revealed 1574 tentative genes that likely were activated and 223 tentative genes repressed directly by Zfp322a. (**D**) Predicted binding motifs for Zfp322a. Motifs were computationally determined based on the ChIP-Seq data. Three different motifs were identified, namely motif 1, motif 2, motif 3, with frequencies of 9%, 5%, 4% respectively. (**E**) Zfp322a can be integrated within ES cell transcription regulatory network. Shown was co-occurrence of transcription factors at the multiple binding loci. Colours in the heat map reflected the co-localization frequency of each pair of transcription factors (the darker the color was, the more frequently colocalized). All the transcription factors were clustered according to the colocalization frequency with other factors, which was calculated based on their co-occurrence at the same binding loci. (**F**) Zfp322a can interact with Oct4. Cell lysate of wild type ES cells were immunoprecipitated using either anti-ZNF322A antibody or anti-Oct4 antibody. Western blot was subsequently carried out with anti-Oct4 antibody or anti-ZNF322a antibody. Control IP was performed using anti-IgG antibody.

The location of the binding site within the gene was mapped as well (Figure 4A). Notably, after putative Zfp322a binding sites were mapped to nearest genes, 62% fall within the TSS of the nearest to gene, showing an obvious preference for TSSs (Figure 4B). 19% of the loci were within gene intronic regions, followed by 5′ UTR, distant promoter (>3 kb from TSS) and promoter (<3 kb from TSS) which occupied 6% each (Figure 4B). Thus, we proposed that Zfp322a is primarily associated with gene promoters.

Among highly enriched binding sites-associated genes, there were many known key components of ES transcription regulatory network, such as *Ino80d*, *Zfp206*, *Zfx*, *Nrf1*, *Smarce1*, implying that Zfp322a could directly regulate transcription of these pluripotency genes (Table S3). To further examine whether Zfp322a targets have preferentially any particular biological functions in mES cells, genes associated with the putative binding sites were subjected to Gene Ontology to search for enriched biological process terms. Large numbers of terms were found to be related to cellular metabolic and biosynthetic processes. Other enriched terms were classified into function groups and summarised in Table S4. Similar to GO analysis of our microarray data, Zfp322a targets were involved in regulation of gene transcription and translation, especially transcription from RNA polymerase II promoter. Notably, Zfp322a binding sites were found near genes encoding core components of RNA polymerase, such as*Polr2a*, *Polr2j*, *Polr3e*. We also found that the targets of Zfp322a were related to developmental processes, implying that Zfp322a may participate in mouse embryo development through regulation of these genes. In addition, many terms surrounding the functions “DNA repair”, “protein modifications”, “cellular component localization”, and “RNA processing”, were enriched.

Besides *Pou5f1* and *Nanog*, we sought to refine our prediction of Zfp322a targets by combining ChIP-seq and microarray data in pluripotent mES cells. We analysed the ChIP-seq in concert with microarray dataset. Overlapping genes between these two sets of data indicated that these genes could be directly regulated by Zfp322a, either positively or negatively. We found that 401 of the 1574 up-regulated genes in the *Zfp322a*-RNAi microarray data analysis were directly repressed by Zfp322a in mES cells, while 223 genes were activated directly (Figure 4C). Further GO analysis of the directly repressed targets, showed that MAPK pathway related genes was enriched (*p* value = 0.006, Table S5). This reaffirmed our hypothesis that Zfp322a represses MAPK signalling pathway to maintain mES cell pluripotency.

### 5. Computational analysis of Zfp322a binding sites

Next, we aimed to identify the Zfp322a binding motif. Through bioinformatic computation, we found three different motifs which repeatedly occurred in Zfp322a binding sites; albeit at low frequencies (Figure 4D). Motif 1 had the highest frequency, presenting in 9% of all the binding sites. Motif 2 was a 12 bp-polyA-sequence with a frequency of 5%. The third motif, which was found in 4% of all binding sites, showed a high similarity to the Oct4/Sox2 binding motif [17]. This suggested that Zfp322a, Oct4 and Sox2 often bind to the same enhancer element, either acting as a complex or interacting in other ways. Notably, the first and the third element are present in the CR4 element of *Pou5f1* and the proximal promoter of *Nanog*. It is expected that the actual Zfp322a binding site was not identified as a consensus binding motif, given that Zfp322a protein harbours 10 zinc fingers, while only 3–5 zinc fingers were needed for specific DNA binding [30]. Therefore, different zinc fingers of Zfp322a possibly recognise distinct sequences, leading to a wide variety of Zfp322a binding motifs.

Given the possibility that Oct4/Sox2 binding sites tended to be present near Zfp322a binding sites, we compared Zfp322a binding sites with target sequences of other transcription factors mapped in previous studies [17]. All the transcription factors were clustered according to the similarity of the co-localization with other factors. The results showed that Zfp322a was closer to the Myc cluster (Figure 4E). But with a closer check of the results, Zfp322a actually had a ubiquitous comparable correlation with all the 12 transcription factors, and did not show any significant preference for either Myc or Oct4/Sox2 centred clusters. Indeed Zfp322a showed a slightly higher co-localization frequency with Oct4 cluster target, from which it was inferred that Zfp322a may facilitate or cooperate with Oct4 in mES cells.

Given the correlation between Zfp322a targets and Oct4 targets, similarities between Oct4/Sox2 and Zfp322a binding motifs, together with our observation that both Zfp322a and Oct4 bound to the CR4 region of *Pou5f1* distal promoter and *Nanog* proximal promoter at the same regions, we sought to determine whether Zfp322a could physically interact with Oct4. Co-IP experiments were performed with either anti-Zfp322a antibody or anti-Oct4 antibody. Western blots were then carried out with anti-Oct4 antibody or anti-Zfp322a antibody. We observed an Oct4 band in Zfp322 IP lane and Zfp322a band in Oct4 IP lane, indicating that Zfp322a physically interacts with Oct4 in mES cells (Figure 4F). This confirmed our hypothesis that Zfp322a functions as a partner of Oct4 in the regulation of gene transcription, though previous studies did not list Zfp322a as an Oct4 partner [31], [32].

### 6. Zfp322a can enhance OKSM reprogramming of MEFs into iPSCs

**Figure 5:**
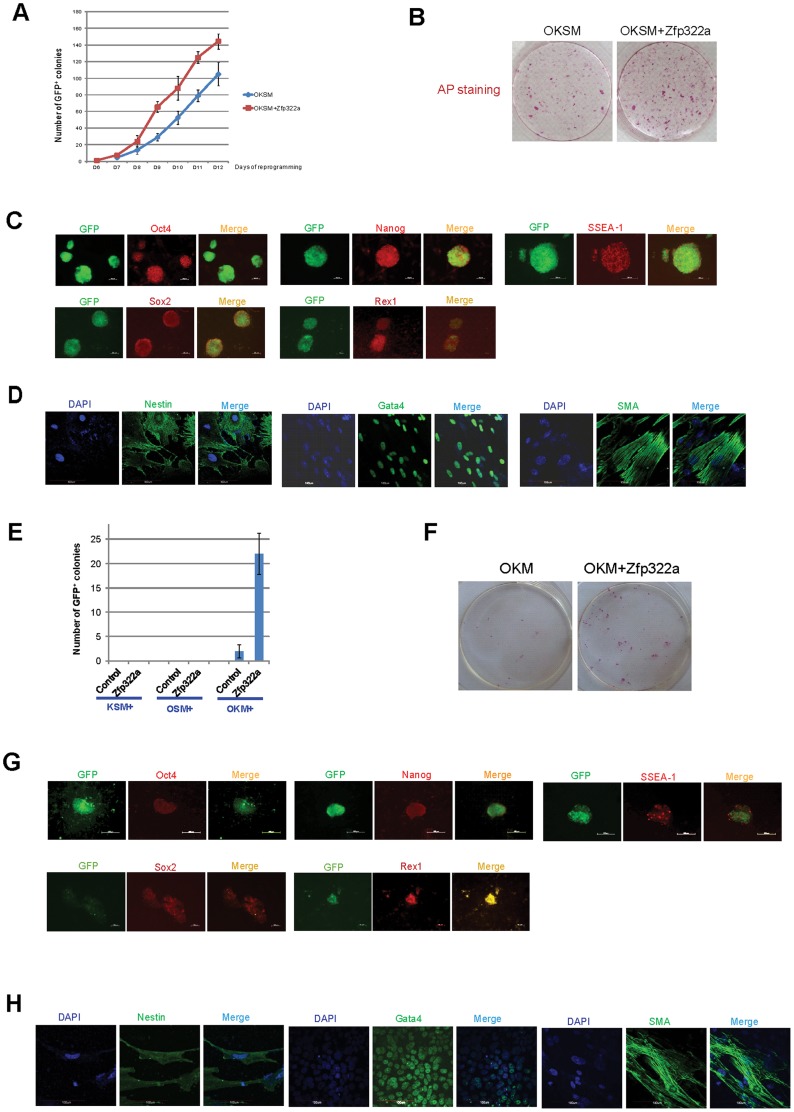
Zfp322a can enhance OSKM reprogramming and replace Sox2. (**A**) Zfp322a enhanced reprogramming efficiency and accelerated the onset of reprogramming process. OSKM serves as control experiment. (**B**) The iPSCs generated from OSKM plus Zfp322a presented alkaline phosphatase activity. There were more AP stained colonies generated from OKSM+Zfp322a compare to OKSM. (**C**) The iPSCs expressed endogenous Oct4, Nanog, Sox2, Rex1 and SSEA-1, indicating that they were ES-cell like. Immunostaining using anti-Oct4, anti-Nanog anti-Sox2, anti-Rex1 and anti-SSEA-1 antibodies were performed with GFP^+^ iPSCs generated from OKSM+Zfp322a. (**D**) GFP^+^ iPSCs generated by OKSM+Zfp322a were able to express ectoderm, mesoderm and endoderm lineage markers in the EB formation assay. iPSCs were stained with anti-Nestin, anti-Gata4 and anti-alpha smooth muscle actin (SMA) antibodies and pictures were taken at 60× magnification. DAPI (blue) served as nucleus marker. (**E**) Zfp322a was able to replace Sox2, but not Oct4 or Klf4 in OSKM reprogramming process. Results from three independent experiments were presented. (**F**) iPSCs generated from OKM plus Zfp322a were positive with AP staining and more AP positive colonies were observed in OKM+Zfp322a as compared to OKSM. (**G**) iPSCs generated by OKM plus Zfp322a expressed pluripotency markers Oct4, Nanog, Sox2, Rex1 and SSEA-1. (**H**) iPSCs derived from OKM+Zfp322a could differentiate into ectoderm, mesoderm and endoderm lineages, which were showed by anti-Nestin, anti-Gata4, anti-SMA staining respectively.

Since Zfp322a is involved in mES cell self-renewal and pluripotency regulation, it would be interesting to investigate whether overexpression of Zfp322a can enhance OSKM reprogramming or act as a novel reprogramming factor to replace any of the OSKM factors in generating iPSCs. MEFs transfected with a *Pou5f1*-GFP reporter were used to identify putative iPSC colonies [33]. It was observed that MEFs infected with OSKM plus Zfp322a showed a more efficient and faster reprogramming process than OSKM alone (Figure 5A). Addition of Zfp322a could enhance the kinetics of OKSM reprogramming as GFP expressing colonies were detected earlier than OKSM control. The number of iPSCs, when counted as GFP^+^ colonies formed by OKSM plus Zfp322a was higher than OKSM throughout the whole reprogramming process. By day 14 of reprogramming process, the number of GFP^+^ colonies generated from OKSM plus Zfp322a was 1.4 fold higher than control. Further examination of these iPSC colonies by AP staining also showed more AP positive colonies formed by OKSM plus Zfp322a as compared to OKSM alone (Figure 5B).

Next, the pluripotency of the iPSCs generated by OKSM plus Zfp322a were evaluated. The expression of GFP suggested that *Pou5f1* promoter was strongly reactivated in iPSCs generated from OKSM plus Zfp322a. IF staining results confirmed that these iPSCs expressed pluripotency markers Oct4, Nanog, Sox2 and Rex1. ES cell marker SSEA-1 was also expressed in the iPSCs generated from OKSM plus Zfp322a (Figure 5C). To further characterize the pluripotency of these iPSCs, embryoid body (EB) formation assays were performed to examine whether these reprogrammed cells were able to differentiate into three germ layers. iPSCs were cultured in suspension to form EBs and then transferred to coated plate with EB differentiation media for 14 days before they were stained with lineage markers. We found that EBs derived from those iPSCs were able to express endoderm marker Gata4, mesoderm marker alpha smooth muscle actin (SMA) and ectoderm marker Nestin (Figure 5D). Therefore, it was demonstrated that iPSCs generated from OKSM plus Zfp322a were pluripotent and closely resembled mES cells. Taken together, we concluded that Zfp322a could induce OKSM reprogramming of MEFs into iPSCs more efficiently and faster.

### 7. Zfp322a can replace Sox2 in the OKSM reprogramming

We then investigated whether Zfp322a can replace the core reprogramming factors in addition to enhancement of reprogramming efficiency. Given that c-Myc is dispensable for reprogramming [34], [35], we only investigated whether Zfp322a can replace any of the OKS factors to generate iPSC colonies from MEFs. Zfp322a was unable to replace Oct4 or Klf4, however, there were GFP^+^ colonies observed in the MEFs infected with Zfp322a plus OKM (Figure 5E). This indicated that exogenous Zfp322a could replace Sox2 in OKSM reprogramming, albeit at a lower efficiency than OKSM. The first GFP^+^ colony generated from Zfp322a plus OKM was observed later as compared to OKSM control. In addition, the expression of GFP was weaker and the number of GFP^+^ colonies was fewer (Figure 5E, 5F, Figure S7A). Similar to iPSCs formed from OKSM plus Zfp322a, these iPSC colonies were positive for AP staining (Figure 5F, Figure S7B). Further examination of the pluripotency profile of these iPSCs showed that these iPS colonies could be stained with anti-SSEA-1, anti-Oct4, anti-Nanog, anti-Sox2 and anti-Rex1 antibodies, and were able to express all three lineage markers when they were induced to differentiate in the EB formation assays (Figure 5G, 5H). These suggested that Zfp322a could replace Sox2, but the combination of OKM plus Zfp322a might have relatively slower kinetics in generating iPSCs than OKSM.

## Discussion

The unique properties of mES cells are governed by the master regulators Oct4, Nanog and Sox2, along with a variety of transcription factors (TFs) [17]. These transcription factors form a complex network to regulate mES cell identity. So far, a lot of TFs have been identified to be important for mES cell identity. However, it is still important to identify novel transcription factors since depletion of a single factor will alter mES cell pluripotency [36], [37]. Our research revealed for the first time that Zfp322a is a transcription factor which is important in maintaining mES cells in undifferentiated state.

Our results have demonstrated that depletion of *Zfp322a* through RNAi induced differentiation of mES cells. The differentiation of mES cells could be due to the suppression of *Pou5f1* and *Nanog* expression after *Zfp322a* was depleted. Zfp322a was shown to actively regulate *Pou5f1* transcription through binding to the CR4 region, and activate *Nanog* transcription via *Nanog* MTL, which are the co-binding sites of other pluripotency factors. Our ChIP-seq and microarray analysis further revealed the binding of Zfp322a to the locus of many other key pluripotency genes that were down-regulated upon *Zfp322a* depletion. It is interesting that Zfp322a can regulate *Pou5f1* and *Nanog* transcription, while Zfp322a itself may also be a target of Oct4 and other pluripotency genes (Figure S1). It appears that these pluripotency factors can form a regulatory loop within the transcriptional network controlling the pluripotency of mES cells. Thus it can be inferred that Zfp322a is a regulator of mES cells by targeting or possibly cooperating with other pluripotency factors.

Moreover, our gene ontology analysis of ChIP-seq and microarray data strongly suggested that Zfp322a targets are involved in embryonic development process and related pathways. Zfp322a is implicated as a pluripotency factor because of its relatively high expression in ICM and undifferentiated mouse ES cells. Since the general reduction trend of Zfp322a expression correlates with a commitment to differentiation and a transient up-regulation of *Zfp322a* at third day of differentiation possibly indicates a specific role of Zfp322a in regulating early lineage commitments. We speculate that Zfp322a not only simply maintains mES cell in their undifferentiated state but also has certain roles in lineage specifications in embryo development. However, these would warrant further studies.

Notably over-expression of Zfp322a in mES cells did not significantly change cell morphology (Figure S8A). Interestingly, *Nanog* level was activated via Nanog proximal promoter in Zfp322a over-expressing mES cells, while *Pou5f1* level showed no significant change over control (Figure S8B, C). Nanog is a well-known core regulator of ES cells, which can sustain ES cell pluripotency in the absence of LIF [27], . The activation of *Nanog* was also observed in a lot of other pluripotency factors, such as Zfp296, Nr5a2, Zic3 etc. [12], [39], [40]. All of these factors are required for maintaining ES cells in their undifferentiated state and can induce *Nanog* expression when over-expressed in ES cells. Moreover, our microarray data and ChIP-seq results showed that Zfp322a can repress MAPK/ERK pathway. It is highly possible that Zfp322a, when over-expressed, may serve as an ERK pathway repressor which results in elevated Nanog expression, mimicking high and homogeneous *Nanog* expression in “2i+LIF” ES culturing media containing ERK inhibitors [20], [24], [25]. Therefore we hypothesize that Zfp322a can activate Nanog expression either directly or indirectly via MARK/ERK pathway inhibition to maintain the ground pluripotency in mES cells.

In our results, we also discovered the interaction between Oct4 and Zfp322a and presence of a similar binding motif for Zfp322a and Oct4/Sox2. This is in concert with the observed higher co-occurrence frequency between Oct4 and Zfp322a binding loci based on the ChIP-seq analysis. Indeed, when we compared the gene expression profile changes after *Zfp322a* RNAi with *Pou5f1* RNAi, a large number of high overlapping targets were identified (Figure S9) [8]. Gene ontology analysis of these co-targeting genes displayed a large number of terms related to cellular, organic, embryonic development, cell proliferation and apoptosis, chromatin remodelling, DNA transcription *etc.* (Table S6). In addition, many Oct4-interacting proteins were also affected in the microarray data analysis (Table S7). These implied a close correlation between Zfp322a and Oct4.

Furthermore, the observation of replacement of Zfp322a to Sox2 in OKSM reprogramming verified Zfp322a facilitation of Oct4 functions. Sox2 was discovered as a transcription factor that binds next to Oct4 motif, thus acting synergistically to active gene transcriptions [8], [9]. But subsequent studies have indicated that Sox2 functions redundantly in the activation of Oct-Sox element [41]. It is then suspected that Zfp322a could have roles partially redundant with Sox2 to interact with Oct4 and participate in Oct4/Sox2 gene regulations. Besides, it is noteworthy that Zfp322a knock-down also depleted expression of *Sox2*, which suggested that Zfp322a is required for *Sox2* activation (Figure 1D, 1E). Therefore, Zfp322a may play a similar function of Sox2 or activate Sox2 expression to mediate its functions.

However, as a Krüppel-like zinc finger transcription factor, Zfp322a may have more complex roles in mES cells since these zinc finger transcription factors have evolved to fill roles in many different biological processes [42]. Indeed, our further analysis of genome-wide Zfp322a target sequence revealed that Zfp322a displayed a non-preferential consensus sequence binding and ubiquitously co-localized with other key pluripotency regulators. Therefore, the ubiquitous association with other factors and diverse implicated functions from microarray and ChIP-seq analysis render Zfp322a functions more complicated. Given that Zfp322a protein contains 10 zinc finger motifs, it might associate with a wide variety of co-factors through different fingers, and therefore its function is depending on the co-factors it interacts with. This pattern may be similar to other C2H2 Zfps harbouring multiple zinc fingers [43], [44]. Taken together, we hypothesize that Zfp322a function as a coordinator that fine tunes the association and recruitments of various factors, including Oct4. It would be interesting to further examine the association of Zfp322a with other components of Oct4/Sox2 regulatory cluster and also Myc cluster proteins to examine their interactions.

Considering the relatively low efficiency and considerable time for the OKSM reprogramming process, many studies focused on finding new factors or developing new methods that can accelerate the kinetics of reprogramming process or defining the reprogramming mechanism. Our results from iPSC formation assays identified Zfp322a as a novel reprogramming enhancer that can replace Sox2, thus expanding the current reprogramming code. Addition of Zfp322a was shown to induce OKSM reprogramming more efficiently and faster than OKSM alone, suggesting that Zfp322a, as a partner of Oct4 and a key regulator of mES pluripotency, can accelerate and enhance the efficiency of this process. To our understanding, Zfp322a seems to have a more remarkable role in increasing the portion of GFP^+^ colonies than increasing the number of AP^+^ colonies. Since the expression of GFP indicates the reactivation of ES cell marker *Pou5f1*, this implies that Zfp322a could facilitate the transition of partially reprogrammed AP^+^ colonies towards fully reprogrammed GFP^+^ colonies.

There are several possible ways in which Zfp322a can enhance reprogramming. Firstly, overexpression of *Zfp322a* has been shown to activate *Nanog* expression. Although Nanog is not one of the canonical quartets of transcription factors used for reprogramming, it is essential for the transition from dedifferentiated intermediates to ground state pluripotency [45]. Thus the enhancement and acceleration of reprogramming brought by Zfp322a could be partially facilitated by Nanog induction. This may also be the same mechanism shared by Nr5a2 and Zfp296 in their enhancement of reprogramming efficiency [12], [40]. Secondly, reprogramming process consists of down-regulation of lineage specific markers, activation of ES cell genes and widespread chromatin remodelling to re-establish the unique chromosomal confirmation of ES cells. As mentioned previously, Zfp322a was shown to repress the lineage specific markers and act as an activator for mES pluripotency genes (Figure 1). ChIP-seq analysis revealed that Zfp322a has many targets involved in chromosome assembly and modifications. Recruitment of epigenetic modifiers, such as histone acetyltransferases, and inhibition of DNA methytransferases and histone deacetylases, can promote reprogramming by loosening the condensed chromatin and thus enabling the exogenous reprogramming factors to access and transcribe pluripotency genes and jumpstart the pluripotency transcriptional network. Therefore, Zfp322a could possibly aid in reprogramming by activating directly or establishing a permissive chromatin state to allow the transcriptions of mES cell-specific genes. Thirdly, Zfp322a may promote the reprogramming via facilitating Oct4/Sox2 functions. Given our observation that Zfp322a is an interacting partner of Oct4 that can replace Sox2 in the OKSM reprogramming, it can be inferred that Zfp322a has similar functions of Sox2. Fourthly, the suppression of MAPK/ERK pathway is implicated in the predicted Zfp322a direct repressed targets. Although the total ERK level was not affected, the elevated p-ERK level upon *Zfp322a* RNAi indicates that Zfp322a could repress ERK pathway but not ERK expression. ERK pathway has been shown to trigger mES cell differentiation [46]. Inhibition of this pathway is important for maintaining the ground pluripotent state of mES cells and can improve somatic cell reprogramming efficiency as well [21], [47]. Thus Zfp322a could also possibly enhance the reprogramming efficiency via the suppression of MAPK/ERK cascade. Therefore, Zfp322a can maintain mES cell properties and promote reprogramming process in many aspects, yet the underlying mechanisms warrant further investigations.

Although human and mouse ES cells are differed in the signaling networks and epigenetic landscapes, it has been revealed that they share the same core regulators Oct4/Sox2/Nanog and similar transcriptional regulatory network, and the well-known Yamanaka factors OKSM are able to drive reprogramming of both human and mouse somatic cells [6], [48], [49], [50]. Given that Zfp322a is a conserved zinc finger protein in human and mouse [16], we propose that Zfp322a is extremely possible to have similar functions regarding the maintenance and acquaintance of pluripotency in human cells, which is very worthy to be elucidated in future studies.

## Materials and Methods

### Cell culture

Murine ES cells (E14) were cultured in ES cell medium consisting of Glasgow Minimum Essential Medium (GMEM; Invitrogen), 15% ES cell qualified fetal bovine serum (FBS; Invitrogen), 0.055 mM β-mercaptoethanol (Sigma), 100 mM sodium pyruvate (Invitrogen), 0.1 mM MEM nonessential amino acid (NEAA, Invitrogen) and 1,000 units/ml of LIF (Millipore).

Platinum-E (Plat-E) cells were maintained in Plat-E medium consisting of Dulbecco's Modified Eagle Medium (DMEM; Invitrogen) containing 10% FBS, 1% penicillin and streptomycin. For cells transfected with retroviruses, medium were supplemented with 1 ug/ml puromycin (Sigma-Aldrich) and 10 ug/ml blasticidin (Invitrogen).

SNL feeder cells were maintained in GMEM, 10% FBS and 1% P/S. Medium was changed every 2 days and cells were passaged every 2–3 days. Inactivated SNL feeder cells were prepared by adding Mitomycin C solution (12 µg/ml, Sigma) to cell culture medium and cells were left to incubate for 2.5 h in 37°C with 5% CO_2_ incubator. The inactivated cells were then passaged and seeded at 80% confluence for culturing iPS cells.

*Pou5f1*-*GFP* MEFs were cultured in mESC medium without LIF. For iPSC formation, MEFs that have been infected with retroviruses were maintained in mESC medium without LIF till 5 days post infection and then maintained in Knockout Serum Replacement (KSR) medium. KSR medium contains DMEM, 15% KSR (Invitrogen), 2 mM L- Glutamine (PAA), 1 mM sodium pyruvate, 1000 units/ml of LIF, 1% P/S, 0.055 mM β-mercaptoethanol and 0.1 mM MEM NEAA. All the cells were cultured in 37°C with 5% CO_2_ incubator.

### Plasmid construction

For RNAi design and construction of plasmids for shRNA synthesis, 19 base-pair gene-specific regions were designed. Oligonucleotides were cloned into pSuper.puro (Oligoengine). All sequences were analysed by BLAST to ensure specificity. For overexpression studies, full-length *Zfp322a* (NM_172586.3) was amplified by PCR and inserted into BamH1 and Xho1 site of pPyCAGIP.

For plasmids used in luciferase assays, *Pou5f1* CR4 region and CR1 region was amplified and cloned into the pGL3-Promoter vector (Promega) upstream of the firefly luciferase gene to give the *Pou5f1* CR4-pSV40-*Luc* and *Pou5f1* CR1-pSV40-*Luc* luciferase reporter plasmids respectively; *Nanog* proximal promoter was amplified and inserted into pGL3-Basic vector to generate the p*Nanog* PP-*Luc* plasmid.

For retrovirus packaging plasmids, full-length *Zfp322a* cDNA was amplified by PCR and ligated into MunI and NotI restriction sites of pMX plasmid (Addgene).

The primers being used are available in the Supplementary Table S8.

### Transfection, RNA extraction, reverse transcription and quantitative real-time PCR

Transfection was conducted using Lipofectamine 2000 (Invitrogen) according to the manual provided. For shRNA, cells were selected in 6-well culture plate for 3 d using puromycin. ES cells transfected with overexpression vectors were selected using puromycin for 1 week before transferring to 100 mm plate for further selection for another 1 week. Single colonies were picked up and passaged to 6-well dishes. The cells were then harvested for either extraction of protein or RNA. RNA extraction, reverse transcription and qPCR for examine of gene expressions were conducted according to previous studies [51]. For ChIP experiments, relative occupancy values were calculated by determining the apparent IP efficiency (ratios of the amount of ChIP enriched DNA over that of the input sample) and normalized to the level observed at a control region. All the qPCR primers are available in Supplementary Table S8.

### Microarray analysis

E14 cells were transfected as described above, with plasmid expressing shRNA targeted against either *Zfp322a* or control. Cells were harvested after 4 days selection. Total RNA was extracted and purified as described above. Then the RNA was diluted to 200 ug/ul and was analyzed using Affymetrix Mouse Genome MG430 Plus 2.0 Array according the manufacture's instruction.

Microarray data was processed to extract the representative intensities from each probe set using RMA [52]. Fold change of 1.5 was used to identify differential expression between the two sample groups (knockdown versus wild type), and only differentially expressed genes were subjected for further analysis. Prior to hierarchical clustering, log2 transformation was first performed and the transformed data were subtracted from the mean of the means of the two sample groups. To identify the enriched “Gene Ontology” (GO) terms in the differentially expressed genes, the GO TermFinder [53] was applied. For presentation of enriched KEGG pathways in the differentially expressed genes, the GATHER [54] was used. The p value cut-off of 0.05 was employed for both significant enriched GO terms and KEGG pathways. For overlapping genes between ChIP-seq predicted targets and Microarray altered gene targets, two sets of genes were analysed using VLOOKUP functions in Microsoft Excel. The microarray data has been deposited to NCBI Gene Expression Omnibus (GEO) with accession number GSE45139.

### ChIP-seq

Chromatin was prepared using E14 cells as previously described [55]. Sonicated chromatin was incubated with Protein G magnetic Dynabeads (Invitrogen) coated with 40 µl of anti-ZNF322 antibody (sc-102205, Santa Cruz) overnight. The beads were then washed and Elution buffer (50 mM Tris-HCl pH 8.0, 10 mM EDTA, 1% SDS) was added to the beads and incubated for 45 min at 68°C with agitating at 1400 rpm. The eluent was decrosslinked by pronase. Lastly, DNA was precipitated and dissolved in nuclease free water for real-time PCR.

For ChIP-seq, ChIP DNA library was prepared by utilizing the ChIP-seq Sample Prep Kit (Illumina). Sequencing was then performed using the Genome Analyzer IIx (Illumina) and reads were mapped to the M. musculus genome assembly mm9.

### ChIP-seq data analysis

ChIP-seq peak detection was performed using Partek software with an average fragment size of 300 bps and 0.05 as the cutoff p-value of Mann-Whitney U test for the separation of forward and reverse reads in a peak. In fact, the Partek software combined several methods of fragment size estimation [56], peak identification [57] and peak filtering using the Mann-Whitney U test. We further enriched the peaks by using the fold change of Zfp322a peak heights to IgG peak heights (fold change 3 as cut-off), and a minimal Zfp322a peak height at 9 reads as a further cut-off criterion (Figure S6). The final list of the inferred peaks was subjected to Zfp322a motif finding. MEME-ChIP in the MEME suite (http://meme.nbcr.net/meme/cgi-bin/meme-chip.cgi) was applied to the inferred peaks. Clustering of Zfp322a with other transcription factors (TFs) was used to evaluate the similarity of the TF targeting. The co-localization between the TFs was first computed and the correlation coefficients between each pair of co-localization vector were then determined. With the completion of all pair-wise correlation, a correlation matrix was obtained. With the matrix, a heatmap reflecting the hierarchical clustering of the correlation coefficients was generated. The Zfp322a ChIP-seq data has been deposited to NCBI Gene Expression Omnibus (GEO) with accession number GSE45138.

### Western blot

Western Blot was carried out as previously described [51]. Primary antibodies used were: anti-ZNF322 antibody (sc-102205, Santa Cruz), anti-β-actin (sc-81178, Santa Cruz), anti-Oct4 (sc-8628, Santa Cruz), and anti-Nanog (sc-33760, Santa Cruz), anti-Sox2 (sc-99000, Santa Cruz), anti-Rex1 (sc-377095. Santa Cruz), anti-p-ERK (sc-7383, Santa Cruz), anti-t-ERK (137F5, Cell Signaling).

### Immunofluorescence staining

Cells cultured in 24-well dishes were fixed in 4% paraformaldehyde and permeabilized with 0.25% Triton X-100, followed by blocking with 3% BSA in PBS. Then cells were probed with primary antibodyin 3% BSA for 1 h at 4°C and secondary antibodyin 3% BSA for 30 min at room temperature. A drop of Vectashield mounting medium with 4′, 6-diamidino-2-phenylindole (DAPI; Vector Laboratories) was placed on the microscope slide and the cover slip was sealed with nail polish in a way that the ES cells were in contact with the mounting medium. Staining signal was then observed through the Axio Observer A1 inverted light microscope (Zeiss). Primary antibody used were: anti-Oct4 (sc-8628, Santa Cruz), and anti-Nanog (sc-33760, Santa Cruz), anti-Sox2 (sc-99000, Santa Cruz), anti-Rex1 (sc-377095, Santa Cruz), anti-SSEA-1 (mab34301, Millipore), anti-alpha smooth muscle Actin (ab5694, Abcam), anti-Nestin (mab2736, R&D), anti-Gata4 (sc-25310, Santa Cruz).

### Flow cytometry

Cells were collected by centrifugation and resuspended in PBS with 4% formaldehyde for 10 min at 37°C. After chilled on ice for 1 min, the cells were resuspended in 90% methanol. The cells were then incubated on ice for 30 min for permeabilization. The cells were rinsed with incubation buffer (0.5% BSA in PBS). The cells were incubated with primary antibody overnight at 4°C. The cells were rinsed twice and then incubated with secondary antibody for 1 h at room temperature in darkness. The cells were rinsed twice before analysed on the flow cytometer (BD FACSCanto). The flow cytometry results were analysed with flow software 2.5.0.

### Dual-luciferase assays

Gene-specific shRNA (600 ng) was cotransfected with *Pou5f1*CR4-*Luc* reporter (600 ng), *Pou5f1* PP-*Luc* or *Nanog* pp-*Luc* reporter (600 ng) and an internal control pRL-TK (30 ng, Promega) encoding *Renilla luciferase*. *Firefly* and *Renilla luciferase* activities were measured with the dual-luciferase reporter system (Promega) 72 h post-transfection by Ultra 384 Microplate Reader (Tecan). The data generated from gene-specific shRNA cells were expressed as relative to non-targeting shRNA control transfection, after normalization to *Renilla luciferase* readings. Transfections were performed in duplicate and on three independent occasions.

### Alkaline phosphatase (AP) staining

AP staining was performed using Alkaline Phosphatase Detection Kit (Millipore) according to manufacturer's instructions and results were obtained using the Axio Observer A1 inverted light microscope (Zeiss).

### Retrovirus packaging and infection

Plat-E cells were seeded onto a 10 cm tissue culture plate at 50–70% confluency and transfected with specific retrovirus packaging plasmids 4–6 h later. Transfection was performed as normal RNAi assays but in this experiment, 24 µg of plasmid, 60 µl of Lipofectamine 2000, and 1.5 ml of Optimen (Invitrogen) were used instead. Cells were incubated overnight in a 37°C, 5% CO_2_ incubator before changing to fresh medium. Virus-containing medium was collected 48 h post transfection, filtered using a 0.22 µm cellulose acetate filter (TPP) and concentrated 100× using Amicon Centrifugal Filter Units-100 kDa (Millipore) by centrifugation at 3800 rpm for 45 min. The concentrated viruses were stored in −80°C for infection use. *Pou5f1-GFP* MEFs were seeded onto a gelatin-coated 24 well plate at 50–70% confluency 6 h before infection. 10 ul of each concentrated retrovirus, supplemented with 8 µg/ml polybrene (Sigma), were added to the MEF cells. MEFs were then passaged onto the inactivated feeder layer 2 dpi and maintained in mESC medium without LIF before replacing with KSR medium at 5 dpi. KSR medium was replaced every day and appearance of GFP^+^ colonies was observed.

### EB formation assay

iPSCs were treated with 0.05%Trypsin/EDTA and then cultured in ES medium without LIF in Ultra Low Culture Dish (Corning) for 5 days. Then the EB were transferred to gelatin-coated 24-well plate and cultured for 14 days. Immunostaining were performed with antibodies for specific lineage markers. Images were captured under a confocal microscope (Olympus FV1000) at 60× magnification.

## Supporting Information

Figure S1 *Zfp322a* intronic region is bound by multiple transcription factors. (**A**) The position of putative binding sites was shown. The black box represents the amplified product from the primer pairs along the intronic region. Open boxes represent exons of *Zfp322*. (**B**) Oct4 binds to *Zfp322a* intronic region. ChIP DNA with anti-Oct4 antibody was analyzed by qPCR. Fold enrichment was obtained after comparing the values of ChIP DNA to that of the input DNA and normalized against a control region.
(EPS)

Figure S2 Zfp322a in mES cells. (**A**) Zfp322a is distributed in nucleus and cytoplasm in ES cells. ES cells were stained with anti-ZNF322 antibody (green). DAPI (blue) served as nucleus marker. (**B**) anti-ZFP322A antibody is specific to Zfp322a protein. E14 cell lysate was prepared for western blot assay.
(EPS)

Figure S3 (**A**) Validation of gene expression microarray analysis of *Zfp322a* RNAi. Eleven down-regulated genes and 10 up-regulated genes were selected from microarray analysis. ES cells transfected with empty pSUPER.puro vector were used as a control and gene expression levels were normalized against *β-actin*. Relative expression level of each gene in qPCR was compared to microarray analysis results. (**B**) ERK inhibitors could not rescue the down-regulated pluripotency genes in *Zfp322a* depleted cells. (**C**) ERK inhibitors could bring down the up-regulated endoderm and ectoderm lineage markers in *Zfp322a* knocked-down cells. Mesoderm markers were elevated with the addition of ERK inhibitors.
(EPS)

Figure S4 Relative luciferase activities were down-regulated upon *Zfp322a* RNAi, *Pou5f1* RNAi and *Zfp322a* & *Pou5f1* double RNAi using pSV40-*Luc*-CR4 construct (**A**) and pSV40-*Luc*-*Nanog* PP construct (**B**). Two sample t-tests were performed with SPSS software for statistical analysis. * indicated p value<0.05.
(EPS)

Figure S5 Zfp322a is required for ES cell pluripotency in HM1 cells. (**A**) Knockdown of Zfp322a drove ES cells differentiation. Pluripotency genes *Pou5f1*, *Nanog*, *Sox2* and *Rex1* were down-regulated upon *Zfp322a* depletion. (**B**) Zfp322a binds to *Pou5f1* distal enhancer CR4. (**C**) Zfp322a binds to *Nanog* proximal promoter region. (**D**) *Zfp322a* RNAi repressed luciferase activities via CR4 and *Nanog* proximal prompter in HM1 cells.
(EPS)

Figure S6 Validation of ChIP-seq data to determine fold change threshold. Genomic loci harbouring peaks with various fold changes were randomly selected from the ChIP-seq data and categorized into three groups: peak height with 9, 11 or more (>11). These selected loci were validated using qPCR. The resultant enrichment fold were shown in the vertical axis of the graph.
(EPS)

Figure S7 Zfp322a can replace Sox2 in the reprogramming process. (**A**) iPSCs generated from OKM plus Zfp322a expressed weak GFP. (**B**) iPSCs generated from OKM+Zfp322a were AP positive.
(EPS)

Figure S8 Zfp322a overexpressing ES cells maintain undifferentiated state. (**A**) Zfp322a overexpressing cells maintained ES cell morphology and were AP positive. (**B**) Zfp322a overexpressing cells displayed elevated Nanog expression. ES cells transfected with empty pPCAGIP vector were used as a control and gene expression levels were normalized against beta-actin. (**C**) Zfp322a activates Nanog expression via Nanog proximal promoter. Dual luciferase assay were performed using pSV40-*Luc*-*Nanog* pp construct in control (empty pPyCAGIP vectors transfected) and Zfp322a overexpressing cells. Renilla luciferase vector was transfected simultaneously and relative luciferase activities were normalized against Renilla luciferase activity.
(EPS)

Figure S9 Zfp322a may synergize Oct4 in maintaining ES cells pluripotency. Zfp322a share many targets with Oct4. Genes that displayed altered expression levels in gene expression microarray analysis upon *Zfp322a* RNAi were compared to genes altered upon *Oct4* RNAi in previous study.
(EPS)

Table S1 Gene onthology analysis of altered genes in gene expression microarray analysis after *Zfp322a* RNAi (p<0.05).
(XLSX)

Table S2 Enriched KEGG pathways for up-regulated genes in gene expression microarray analysis after *Zfp322a* RNAi (p<0.05).
(XLSX)

Table S3 One hundred binding sites with top-ranked peak heights in Zfp322a ChIP-seq analysis.
(XLSX)

Table S4 Representative enriched gene ontology terms in ChIP-seq targets.
(XLSX)

Table S5 Enriched KEGG pathways for up-regulated direct targets predicted by gene expression microarray and Chip-seq analysis (p<0.05).
(XLSX)

Table S6 Gene ontology results of overlapping genes in the gene expression microarray analysis of *Zfp322a* and *Oct4* RNAi.
(XLSX)

Table S7 List of Oct4-interacting proteins whose encoding genes were altered upon *Zfp322a* RNAi.
(XLSX)

Table S8 Sequences of primers.
(XLSX)
